# CLINICAL TRIALS IN GEROSCIENCE

**DOI:** 10.1093/geroni/igz038.2986

**Published:** 2019-11-08

**Authors:** Matt Kaeberlein, Matt Kaeberlein

**Affiliations:** University of Washington, Seattle, Washington, United States

## Abstract

This session will focus on clinical trials in geroscience.

